# Associations between repetitive negative thinking and resting-state network segregation among healthy middle-aged adults

**DOI:** 10.3389/fnagi.2022.1062887

**Published:** 2022-12-15

**Authors:** Cristina Solé-Padullés, Gabriele Cattaneo, Natalie L. Marchant, María Cabello-Toscano, Lídia Mulet-Pons, Javier Solana, Núria Bargalló, Josep M. Tormos, Álvaro Pascual-Leone, David Bartrés-Faz

**Affiliations:** ^1^Department of Medicine, Faculty of Medicine and Health Sciences; Institute of Neurosciences, University of Barcelona, Barcelona, Spain; ^2^Institut d’Investigacions Biomèdiques August Pi i Sunyer (IDIBAPS), Barcelona, Spain; ^3^Institut Guttmann, Institut Universitari de Neurorehabilitació adscrit a la UAB, Barcelona, Spain; ^4^Division of Psychiatry, University College London, London, United Kingdom; ^5^Magnetic Resonance Image Core Facility, Institut d'Investigacions Biomèdiques August Pi i Sunyer (IDIBAPS), Barcelona, Spain; ^6^Hinda and Arthur Marcus Institute for Aging Research, Hebrew SeniorLife, Harvard Medical School, Boston, MA, United States

**Keywords:** repetitive negative thinking, resting-state networks, system segregation, depression, anxiety, rumination

## Abstract

**Background:**

Repetitive Negative Thinking (RNT) includes negative thoughts about the future and past, and is a risk factor for depression and anxiety. Prefrontal and anterior cingulate cortices have been linked to RNT but several regions within large-scale networks are also involved, the efficiency of which depends on their ability to remain segregated.

**Methods:**

Associations between RNT and system segregation (SyS) of the Anterior Salience Network (ASN), Default Mode Network (DMN) and Executive Control Network (ECN) were explored in healthy middle-aged adults (*N* = 341), after undergoing resting-state functional magnetic resonance imaging. Regression analyses were conducted with RNT as outcome variable. Explanatory variables were: SyS, depression, emotional stability, cognitive complaints, age and sex.

**Results:**

Analyses indicated that RNT was associated with depression, emotional stability, cognitive complaints, age and segregation of the left ECN (LECN) and ASN. Further, the ventral DMN (vDMN) presented higher connectivity with the ASN and decreased connectivity with the LECN, as a function of RNT.

**Conclusion:**

Higher levels of perseverative thinking were related to increased segregation of the LECN and decreased segregation of the ASN. The dissociative connectivity of these networks with the vDMN may partially account for poorer cognitive control and increased self-referential processes characteristic of RNT.

## Introduction

Repetitive Negative Thinking (RNT) is a psychological construct associated with persistent negative thoughts about the future (worry) and past (rumination), which characterizes psychopathological conditions such as anxiety and depression (McLaughlin et al., 2007; Spinhoven et al., 2018). RNT also relates to low self-perceived cognitive functioning (Schlosser et al., 2020) as well as increased cognitive decline and neuroimaging Alzheimer’s disease (AD) biomarkers (Marchant et al., 2020).

Brain correlates of RNT have been previously examined, both from a structural and functional point of view. In a recent review, the dorsolateral prefrontal (DLPFC) and anterior cingulate cortices emerged as key regions, albeit with inconsistent associations due to the different nature of studies, including different types of RNT (Demnitz-King et al., 2021). Nonetheless, authors emphasized that it might be more relevant to explore connectivity of large-scale networks rather than attempt to isolate single brain structures. In this regard, a recent meta-analysis explored functional correlates of RNT, taking into account both task-related and resting-state functional magnetic resonance imaging (fMRI, Makovac et al., 2020). Despite the small sample sizes for all these studies, the authors concluded that activation of medial prefrontal cortex and precuneus (regions of the default mode network, DMN) was associated with rumination among clinical samples, as well as the insula, parahippocampus and the anterior cingulate; this latter also emerging as a significant hub within resting-state studies. Interestingly, the insular and anterior cingulate cortices are linked to self-monitoring, saliency detection and emotional processing, with a particularly critical role of the insula in switching activation of the executive control and DMN to facilitate access to attention (Menon and Uddin, 2010).

Nonetheless, there are only few studies investigating functional correlates of RNT at rest among non-clinical samples, and the ones available only considered young participants. Kuhn and colleagues for example used a seed-based approach to examine local connectivity of the prefrontal cortex during resting-state fMRI among young adults, identifying that unwanted thoughts were negatively correlated with the right DLPFC and positively with the left putamen (Kühn et al., 2014). The DLPFC is a relevant brain region associated with inhibition, planning and working memory (Badre and Wagner, 2004; Brunoni and Vanderhasselt, 2014), which contributes to cognitive and attentional control through its connection with the anterior cingulate (Cohen, 2017). Another previous study combining task-related and resting-state fMRI in non-depressed young adults showed that rumination was linked to an alternating function within the entorhinal cortex, which decreased during cognitive demand (task-fMRI) and increased at rest (Piguet et al., 2014). The authors suggested that attention demands would attenuate activity of memory-related areas associated with rumination, thereby suppressing or minimizing the possibility to ruminate. Therefore, when exploring brain connectivity under low or null attentional demands (i.e., resting-state fMRI) it is more likely that tendency to RNT is more manifested by differential connectivity within and between networks linked to self-attention, emotion regulation and cognitive control.

Large-scale resting-state networks are organized in specialized modules, and optimally segregated from one another to ensure successful brain function (Sporns and Betzel, 2016; Wig, 2017). Specifically, connectivity within and between brain networks is balanced in a way that it should promote functional specialization, allowing the successful execution of different processing operations (Sporns, 2013). Graph theory provides a mathematical framework with which neuroscientists can examine the status of different nodes at several levels of complexity (Wig, 2017). Ideally, younger brain nodes exhibit robust relationships with brain regions that belong to the same community system, while interactions with other brain systems are sparser. This concept is referred to as system segregation (SyS) and increasing evidence suggests that segregation of brain networks decreases with age (Chan et al., 2014). Likewise, SyS is associated with clinical decline regardless of AD pathology (Chan et al., 2021). As stated above, RNT involves several brain regions within large-scale networks (Demnitz-King et al., 2021), the efficiency of which depends on their ability to remain segregated (Tononi et al., 1994). As far as we know, there are no studies exploring resting-state networks associated with RNT among non-clinical middle-aged individuals. Ours represents the first large cohort study with middle-aged participants to investigate the associations between the trans-diagnostic cognitive processing style of RNT (Ehring and Watkins, 2008) and segregation of frontoparietal and frontolimbic networks linked to attention, emotion regulation and cognitive control. Anterior Salience and Executive Control networks, apart from the DMN, are target systems in the study of RNT, as their main nodes (anterior cingulate, insula, DLPFC) have been previously linked to modes of thinking, as stated above. Thus, we investigated possible associations between RNT and SyS of the Anterior Salience Network (ASN), Default Mode Network (DMN) and Executive Control Network (ECN) among healthy middle-aged adults.

## Materials and methods

### Subjects

Healthy early and late middle-aged subjects (*n* = 341) were drawn from the Barcelona Brain Health Initiative cohort (Cattaneo et al., 2018). Inclusion criteria were: volunteers older than 40 with minimum Mini-Mental State Examination (MMSE) score of 27. Exclusion criteria included previous history of neurological disease and current or recent (<2 years) diagnosis of psychiatric conditions. Table 1 depicts demographics and other characteristics of the sample. RNT questionnaires and scales exploring mental health were conducted online, and volunteers attended the premises for cognitive evaluations and MRI/fMRI acquisitions. Data for all participants were gathered between October 2018 and early 2020. Informed online consent was obtained in agreement with the Code of Ethics of the World Medical Association (Declaration of Helsinki).

**Table 1:** Demographics and clinical characteristics of the sample (*N* = 341).

Variable	Mean (SD)/Range	Percentage
Age	55.45 (6.66)/43.13–67.93	–
Sex	–	Female 50.1%
Education level	–	Primary: 2.3%, Secondary: 20.2%, University: 77.4%
Mini-Mental State Examination (MMSE)	29.80 (0.53)/27–30	–
Depression Anxiety Stress Scale (DASS)	7.02 (7.61)/0–46	–
Cognitive Complaints (PROMIS)	18.88 (7.64)/11–45	–
Emotional Stability (IPIP-BIG5)	35.23 (8.68)/11–50	–
Rumination Response Scale (RRS)	11.43 (10.73)/0–57	–
Penn State Worry Questionnaire (PSWQ)	8.76 (7.81)/0–32	–
Perseverative Thinking Questionnaire (PTQ)	14.13 (12.05)/0–58	–

### Measures

Following previous studies on the topic, RNT was measured with three main questionnaires, to assess all of its psychological aspects (Schlosser et al., 2020). First, the Perseverative Thinking Questionnaire (PTQ) was administered. Its Spanish validation consists of 15 items based on Ehring’s original version (Ehring et al., 2011) measured in a 5-Likert scale and including questions that try to capture frequency of intrusive thoughts (Ruiz et al., 2020). Additionally, worry was assessed with the Penn State Worry Questionnaire (PSWQ, Hopko et al., 2003; Spanish version by Nuevo et al., 2007), which contains 8 items scored from 1 (not at all typical) to 5 (very typical of me). Finally, rumination was explored with the Rumination Responses Scale (RRS) with its Spanish version by Hervás (2008), including 22 items to be scored in a 4-point Likert scale measuring frequency from 1 (almost never) to 4 (almost always). Cronbach’s alpha for each questionnaire was: PTQ: 0.97, PSWQ: 0.94, and RRS: 0.92. A composite score of the three RNT measures was computed as the sum of the standardized values (z scores) for each questionnaire.

Participants were also asked to complete questions assessing presence of depressive and anxiety symptoms, cognitive complaints, and personality traits. Since RNT may contribute to depression, anxiety and perceived stress (McLaughlin et al., 2007; Spinhoven et al., 2018), we administered the short form of the Depression Anxiety Stress Scale (DASS), which includes 21 items divided into three blocks, to explore these three dimensions (Lovibond and Lovibond, 1995). Cronbach’s alpha for DASS was 0.92 for our sample.

RNT has been associated with lower subjective cognitive functioning and higher memory complaints among healthy older adults (Schlosser et al., 2020), and hence the 12-item PROMIS Cognitive Abilities and Cognitive Concerns Scales was administered to measure subjective perceptions of memory function, attention and planning (Fieo et al., 2016), as explained elsewhere (Delgado-Gallén et al., 2021). The 5-point Likert scale was reversed so that higher scores meant greater cognitive complaints.

Worry and rumination are also considered cognitive processes largely linked to neuroticism (Muris et al., 2009), particularly with rumination having been regarded as a moderator between neuroticism and depressive symptoms, also in non-clinical samples (Roelofs et al., 2008). In this line, our participants were asked to complete the International Personality Item Pool (IPIP-Big5; Goldberg, 1999), whereby a quantitative measure of emotional stability was obtained as an indicator of inverse neuroticism (range of scores between 10 and 50 for each trait).

### Scanning procedure and preprocessing

All participants underwent an MRI session acquired with a 3 T Magnetom Prisma (Siemens Medical Systems, Erlangen, Germany) at the Centre de Diagnòstic per la Imatge of Hospital Clínic (Barcelona, Spain) using a 32-channel radiofrequency head coil. A high-resolution 3D-magnetization prepared rapid acquisition gradient-echo sequence (MPRAGE) and a 10-min-resting-state fMRI were acquired. Parameters are described in the Supplementary section. Images were inspected by a senior neuroradiologist (N.B.) for possible pathologies (none detected).

T1 was acquired in an axial plane with the following parameters: TR = 2,400 ms, echo time (TE) = 2.22 ms, inversion time = 1,000 ms, slice thickness = 0.8 mm and field of view (FOV) = 256 mm and flip angle = 8° (208 total slices). Further, a high-resolution 3D SPACE T2 weighted acquisition was undertaken to optimize anatomic segmentation, with these parameters: TR = 3,200 ms, TE = 563 ms, flip angle = 120°, 0.8 mm isotropic voxel, FOV = 256 mm (208 total slices). Finally, a 10-min resting-state fMRI in an eyes-closed condition was acquired. Subjects were asked not to fall asleep and the following parameters were used: TR = 800 ms, TE = 37 ms, 750 volumes, 72 slices, slice thickness = 2 mm, FOV = 208 mm. All acquisitions were visually inspected before analysis (M.C.-T. and L.M.-P.) to ensure that they did not contain MRI artifacts or excessive motion.

Preprocessing of resting-state images was carried out with FMRIB Software Library,^1^ FreeSurfer^2^ and Statistical Parametric Mapping.^3^ For the purpose of magnetic stability, the first 10 scans were removed. Images were corrected for inhomogeneity-induced distortion with the FSL topup tool. Then, images were realigned to a reference image (FSL MCFLIRT). Functional images were coregistered to native structural image (T1) using the SPM Coregister tool. Finally, all fMRI images were normalized to a standard space (Montreal Neuroscience Institute, MNI152). As regards movement correction, frame-wise displacement was computed for each subject, by using the vectors of rotation and translation obtained during the realign step (Power et al., 2014). Nuisance correction was addressed by manually removing components corresponding to motion regressors (rotation, translation and their derivatives, as estimated during scans’ realignment), a drift estimated by a discrete cosine transform as a low-pass frequency filter (<0.01), and signals from white matter and cerebrospinal fluid. Signal from white matter and cerebrospinal fluid areas was extracted from masks obtained by running the FreeSurfer ‘recon-all’ processing stream. Briefly, this automatic subcortical segmentation of brain volume is based upon the existence of an atlas containing probabilistic information on the location of structures (Fischl et al., 2002). The whole processing stream included default parameters except for the addition of T2-w images to improve pial surfaces reconstruction.

Once fMRI data was preprocessed, blood-oxygen-level-dependent signal was extracted and averaged across all voxels falling within each region of interest (ROI), as defined by the Shirer atlas (Shirer et al., 2012). Then, ROI-to-ROI resting state Functional Connectivity values (rs-FCs) were computed as Pearson-Moment correlations and subsequently Fisher-z transformed. Negative values were set to zero and autocorrelations were not considered in further calculations. Resting-state network segregation and functional connectivity values for each targeted network, namely ECN, DMN and ASN, were computed. Within and between-network connectivity values for each network might be used for post-hoc regression models to further explore the nature of associations linked to system segregation.

SyS, a versatile graph theory-based measure of functional brain integrity, was calculated as expressed in:


$${\mathrm{SyS}}_{\mathrm{net}}=\frac{W_{\mathrm{net}}-B_{\mathrm{net}}}{W_{\mathrm{net}}},$$

${\mathrm{SyS}}_{\mathrm{net}}\;$captures the balance between within-network ($W_{\mathrm{net}})$ and between-networks (B_net_) rs-FC. $W_{\mathrm{net}}$ was computed as the average rs-FC connecting all the nodes within the same network, while $B_{\mathrm{net}}\;$was computed as the average rs-FC connecting nodes of a network to nodes from the rest of the cortex.

### Statistical analysis

A linear regression model was conducted with the RNT composite as the dependent variable, and the following independent variables: age, sex, depression/anxiety/stress (DASS score), emotional stability, cognitive complaints and SyS (for the ASN, ventral and dorsal-DMN, left and right-ECN). We used a stepwise approach and only variables showing an alpha-to-enter significance level lower than 0.05 were retained. Confidence intervals for unstandardized coefficients were set to 99%.

Additional analyses included each RNT questionnaire as a separate outcome to further explore potential different contributions to resting-state network segregation. Finally, the subscale of brooding within the RRS was also taken as a dependent variable, as the scale includes items that explore reflection (adaptive form of rumination style) as well as brooding (negative component of rumination, Hervás, 2008).

## Results

In the main univariate regression model, factors retained and associated with RNT were: depression/anxiety/stress, emotional stability, cognitive complaints, age, as well as SyS of the left ECN (LECN) and ASN. The model explained 73.3% of the variance of RNT, being DASS the factor explaining most of it (adjusted *R*^2^ = 0.656). Thus, depression/anxiety/stress was positively associated with RNT, as well as cognitive complaints. The reverse association was found for emotional stability and age (see Table 2).

**Table 2:** Multiple linear regression analysis with retained explanatory variables, being RNT the predicted variable.

Explanatory variables	Unstandardized coefficients		*t*	Value of *p*	99% CI
	Beta	SE			
Depression	0.21	0.012	17.25	<0.0001	0.18 to 0.25
Emotional stability	−0.73	0.011	−6.67	<0.0001	−0.1 to-0.05
Cognitive complaints	0.04	0.012	3.50	0.001	0.01 to 0.07
Age	−0.03	0.012	−2.84	0.005	−0.06 to-0.003
SyS_LECN	2.41	0.68	3.56	<0.0001	0.65 to 4.16
SyS_ASN	−2.26	0.80	−2.81	0.005	−4.34 to-0.17

SyS, system segregation; LECN, left executive control network; ASN, Anterior Salience Network.

**Figure 1:**
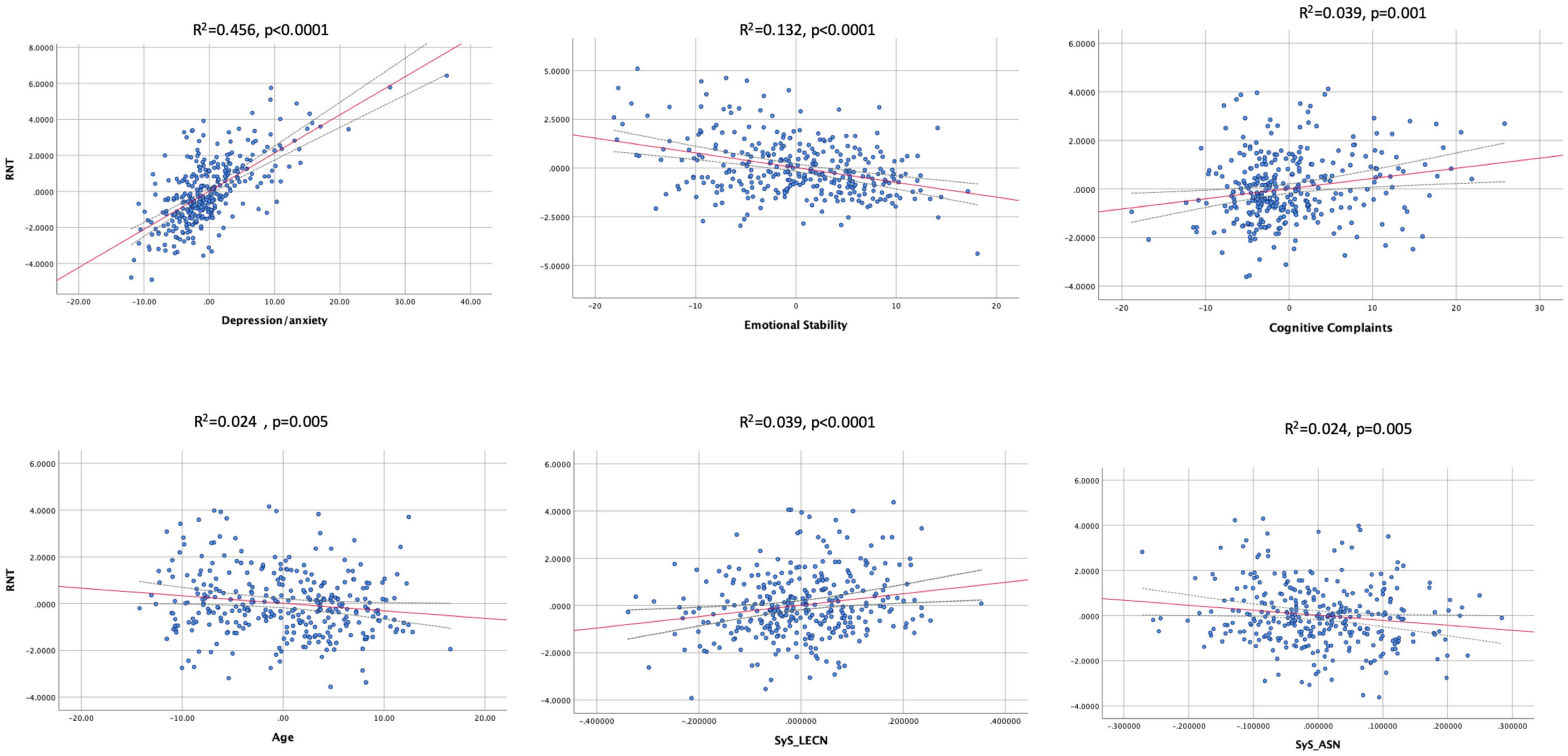
Partial regression plots showing associations between all retained variables and RNT (Repetitive Negative Thinking). Dashed lines indicate the 99% confidence intervals for the regression line. SyS, system segregation; LECN, left executive control network; ASN, Anterior Salience Network.

The association between RNT and network segregation was positive for the LECN and negative for the ASN (see Table 2; Figure 1); therefore, individuals with higher scores of RNT presented more segregation of the LECN, while the ASN was less dedifferentiated from the rest of networks.

Variables not retained after conducting the stepwise regression model were sex and SyS of the DMN (dorsal and ventral) and right ECN.

**Figure 2:**
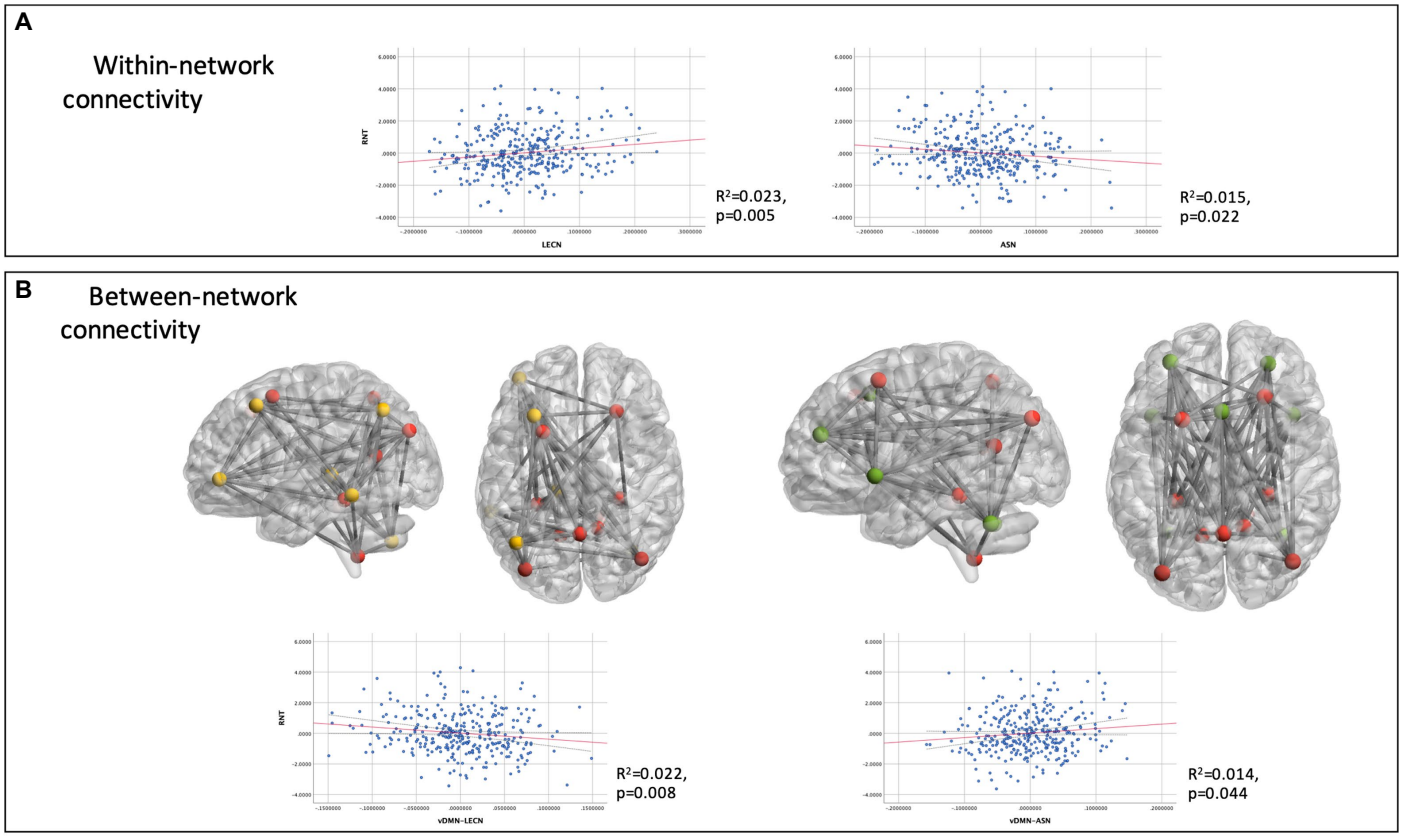
Partial regression plots for within-network connectivity **(A)** and between-network connectivity **(B)** associations with RNT. Dashed lines indicate the 99% confidence intervals for the regression line. For illustrative purposes, glass brains depict connectivity between functional region of interest (ROI) of vDMN (in red) with LECN (yellow) and ASN (green).

A post-hoc regression model was conducted to further investigate within and between-network connectivity, targeting solely on the LECN and ASN. Between-network connectivity included mean functional connectivity values between the targeted networks (LECN and ASN, as well as between themselves and the DMN). Other explanatory variables of the predicted variable (RNT) were: depression, emotional stability, cognitive complaints and age; since they emerged as having a significant effect on the principal regression model. The resulting model explained the 72.3% of the total variance. RNT was associated with increased connectivity within the LECN (Unstandardized beta (b) = 2.63, SE = 0.97; *t* = 2.71, *p* = 0.007; CI 99%: 0.12 to 5.14) and decreased connectivity within the ASN (*b* = −2.12, SE = 0.98; *t* = −2.16, *p* = 0.031; CI 99%: −4.67 to 0.42). Figure 2A depicts within-network connectivity of these two networks relative to RNT scores. Further, connectivity between LECN and the ventral DMN (vDMN), mainly engaging regions of the medial temporal lobe and posterior cingulate cortex, was decreased for those participants with higher scores of RNT (*b* = −3.96, SE: 1.51, *t* = −2.62, *p* = 0.009; CI 99%: −7.88 to −0.04). Conversely, for the connectivity between the ASN and vDMN, the association with RNT was positive (*b* = 2.95, SE = 1.38; *t* = 2.13, *p* = 0.034; CI 99%: −0.64 to 6.54). Figure 2B illustrates the divergent connectivity between vDMN_LECN and vDMN_ASN, as a function of RNT.

As regards analyses using RRS (and brooding subscale), PTQ and PSWQ as separate outcomes, only PTQ was associated with resting-state network segregation variables (LECN and ASN). See Table 3 for statistical details for each model. A post-hoc regression model with only PTQ as the dependent variable showed that only the connectivity within LECN remained still significant (Unstandardized beta (b) = 12.88, SE = 4.81; *t* = 2.68, *p* = 0.008; CI 99%: 0.41–25.35) as did connectivity between vDMN and ASN (Unstandardized beta (b) = 20.49, SE = 6.94; *t* = 2.95, *p* = 0.003; CI 99%: 2.51–38.47).

**Table 3:** Variables entering each regression models conducted for every component of RNT, separately.

Linear regression model: predicted variable	Retained variables	Adjusted *R*^2^	*t*	Value of *p*
RRS (brooding subscale)	1. DASS	0.418	10.71	<0.0001
	2. Emotional stability	0.444	−3.90	<0.0001
	3. Age	0.452	−2.36	0.019
RRS (whole scale)	1. DASS	0.460	11.08	<0.0001
	2. Emotional stability	0.477	−2.34	0.02
	3. Age	0.490	−3.36	0.001
	4. Cognitive complaints	0.502	2.90	0.004
PSWQ	1. DASS	0.481	11.33	<0.0001
	2. Emotional stability	0.572	−8.34	<0.0001
PTQ	1. DASS	0.613	15.53	<0.0001
	2. Emotional stability	0.656	−5.10	<0.0001
	3. Cognitive complaints	0.672	4.17	<0.0001
	4. SyS_LECN	0.677	3.28	0.001
	5. SyS_ASN	0.681	−2.16	0.032

Cumulative adjusted *R*^2^ for each variable retained is presented. DASS, depression anxiety stress scale; SyS, system segregation; LECN, left executive control network; ASN, Anterior Salience Network.

## Discussion

To our knowledge this is the first study to incorporate non-clinical middle-aged participants to study brain functional biomarkers of RNT. Our analyses indicated associations between this psychological construct (particularly PTQ) and segregation of frontoparietal-executive and limbic-related networks, which are linked to cognitive control, attention and emotional regulation. Specifically, participants with higher scores of global RNT presented increased segregation of the LECN and decreased segregation of the ASN. Post-hoc analyses determined that higher segregation of LECN implied increased functional connectivity within itself, while the reverse was found for the ASN. Additionally, the LECN presented decreased connectivity with the vDMN. The ASN was on the other hand more engaged with this posterior component of the DMN. The relevance of DMN and ASN-related regions in perseverative thinking and rumination were already pointed out in the meta-analysis by Makovac et al. (2020). Likewise, the DLPFC and anterior cingulate also emerged as relevant regions in a recent review examining anatomical correlates of RNT (Demnitz-King et al., 2021).

In this line, the LECN mainly encompasses the left DLPFC and left superior parietal cortex and it plays an important role in decision-making, response inhibition, working memory as well as regulation of cognition and behavior (Curtis and D'Esposito, 2003; Aron, 2007; Seeley et al., 2007; Menon, 2011). There is however inconclusive evidence regarding the activity of the DLPFC associated with brooding and rumination. In a recent publication, Borders discusses the role of DLPFC activity in task-related protocols hypothesizing that the increased activity of this region associated with high rumination may be suggestive of overuse of executive resources to overcome the effects of rumination (Borders, 2020). Yet, and as the author pointed out, no data was yet available on functional connectivity within this executive network. Our results indicate more segregation of the LECN and increased within-network connectivity in participants with higher scores of RNT. Hence, LECN is not only more differentiated from the other networks, but the increased intra-network connectivity suggests that more executive resources are being used, also observable at rest, in the context of high perseverative thinking. This additional activity may explain its decreased connectivity with the vDMN. In this line, connectivity between the ECN and the DMN has been associated with autobiographical planning, goal-directed behavior (Spreng et al., 2010) and creative cognition (Beaty et al., 2015). Therefore, it might be sensible to argue that one of the factors contributing to modulate ECN-DMN connectivity is RNT.

On the other hand, the opposite association occurred for the ASN. Specifically, participants with higher levels of RNT presented decreased segregation of this network and therefore the activity within the middle frontal gyrus, anterior cingulate and insula, key regions of the ASN (Shirer et al., 2012), were less differentiated from other networks. This finding was corroborated by the poorer intra-network connectivity of ASN associated with high RNT and increased connectivity with the vDMN. The main hubs of the ASN are the insula and anterior cingulate, both associated with response to salient stimuli, awareness of feelings, empathy and emotional responses (Menon and Uddin, 2010). The vDMN encompasses the precuneus and medial temporal lobe structures; these latter associated with self-referential and autobiographical memories (Bayley et al., 2006). ASN and DMN have a competitive relationship during cognitive processing, being thus both anticorrelated to secure a successful response in the face of cognitive demands (Greicius et al., 2003). Further, the ASN coordinates both the ECN and the DMN, having a critical role in activating the former and deactivating the latter in high demanding tasks (Sridharan et al., 2008; Menon and Uddin, 2010). The combination of worry, rumination and perseverative thinking might introduce a bias in the dependencies among these networks with a decreased connectivity between executive and DMN regions that seems somehow compensated by increased connectivity between ASN-DMN, thus diminishing cognitive control at the expense of increasing self-referential and emotional processes linked to autobiographical memory. This might be particularly true in the absence of attentional tasks or ‘cognitive distractions’ where RNT may become more evident affecting connectivity of memory-related areas, as other authors pointed out (Piguet et al., 2014). Of note, the only RNT component associated with resting-state network segregation was perseverative thinking, measured with the PTQ, a time-independent and unidimensional scale that captures the core characteristics of RNT: repetitiveness, intrusiveness and difficulty to disengage (Ehring et al., 2011).

Primary variables associated with RNT were depression, cognitive complaints and neuroticism. This is of special relevance considering that these are risk factors for dementia (Diniz et al., 2013; Terracciano et al., 2017; Numbers et al., 2020) and modulating RNT might reduce its risk (Marchant and Howard, 2015). The association between RNT and depression/anxiety scores is in line with previous reports suggesting that RNT is a vulnerability factor for the development of mood disorders and constitutes a promising target for prevention (Topper et al., 2017; Spinhoven et al., 2018). It is important to highlight that while our depression/anxiety questionnaire (DASS) explained 65.6% of the total variance of RNT; when the model was reversed and DASS was included as a dependent variable, with the predictors of RNT, sex, age, emotional stability, cognitive complaints and SyS; only RNT emerged as the retained factor to explain most of the variance of depression/anxiety. Hence, while there is substantial overlap between depression and RNT; the former was not related to resting-state network segregation as RNT proved to be in the reverse model, and this verifies that RNT and depression/anxiety should be considered different entities. Therefore, the pattern of differential system segregation for LECN and ASN was exclusively associated with RNT and not with depressive symptoms. This evidence is in line with previous studies concluding that RNT is a larger construct comprising intrusion of thoughts and low levels of mindfulness (Gustavson et al., 2018), apart from common symptoms linked to depression and anxiety.

In our study, RNT was also associated with subjective cognitive complaints and this relationship is also in accordance with previous findings (Schlosser et al., 2020) and reinforced by recent data indicating a connection between RNT and objective cognitive decline (Marchant et al., 2020). Further, the link between RNT and emotional stability (more RNT associated with less emotional stability) has been previously seen in patients with generalized anxiety disorder (McEvoy and Mahoney, 2013), but also among healthy adults (Slavish et al., 2018).

Interestingly, we also found that age was negatively associated with RNT. Thus, the younger middle-aged participants were the ones experiencing higher indices of worry and rumination. Older adults have been hypothesized to be at a higher risk for ruminative thinking, due to the declining executive functions while aging (De Luca et al., 2003). We did not find such an association, although our sample only included 26.7% of subjects aged 60 or above and no participants older than 67. Our result might be partially in line with a previous study by Sütterlin and colleagues, showing that older participants (above 63) reported less ruminative thinking than young and middle-aged adults (Sütterlin et al., 2012). Still, the association between RNT and age remains to be further examined, particularly among the old-old.

In our study, sex was not retained as an explanatory variable for RNT. However, direct measurements of RNT between male and female participants (*T*-test) corroborated increased RNT for the latter (*t* = 2.85, *p* = 0.005). A previous meta-analysis with clinical and non-clinical samples reported that rumination was higher for women adults (Johnson and Whisman, 2013), and was later replicated (Whisman et al., 2020). The fact that sex was not retained in our model indicates that it may not have had enough power to disentangle the effects of sex as compared to the remaining significant effects.

On the whole, the dissociative connectivity between ECN, ASN and DMN linked to higher levels of RNT among healthy middle-aged adults may partially explain poorer cognitive control and increased self-referential processes characteristic of RNT (Piguet et al., 2014). The ECN is of particular importance since goal-directed control processes are driven by thoughts and action (Friedman and Miyake, 2017) and RNT overlaps with executive functions (Gustavson and Miyake, 2016). ASN might regulate emotional and self-referential aspects of RNT by means of its connections with memory-related networks (vDMN). Besides, and as stated above, ASN has a role in switching the activity of the DMN and ECN (Menon and Uddin, 2010) and there is evidence that this interaction operates differently in clinical and non-clinical individuals (Hamilton et al., 2011). Hamilton and colleagues demonstrated that the right fronto-insular cortex (regions associated with the ASN) was active during non-task-related processes in depressive patients (also implying high DMN engagement) and that this was related to maladaptive depressive rumination. Conversely, in healthy subjects, the fronto-insular cortex was active during high attentional demands (meaning low DMN engagement and high ECN activity) and this balance was interpreted as bearing positive and creative psychological processes. Therefore, a good equilibrium of these three networks (DMN, ECN and ASN) seems relevant not only to fulfill cognitive demands but also to enhance other processes possibly linked to adaptive processing and emotional well-being. In our study, we found a pattern of network interaction associated with RNT involving these networks, with a particular association between more perseverative thinking and increased DMN-ASN connectivity, which according to Hamilton’s interpretations is not supposed to be optimal. Further studies are needed to better characterize the association between worry/recurrent thoughts; and possible dysregulated activity of the triad DMN-ECN-ASN.

The present study is not without limitations. First, its cross-sectional approach restricts the interpretation of the associations between RNT and SyS. How RNT associates with resting-state network segregation change across aging and how this might be linked to subjective and objective cognitive decline is of relevance to further address potential therapies directed to reduce perseverative thinking. Second, there was an increased representation of highly educated participants in our cohort. Brain connectivity, particularly within the frontoparietal control network, is acknowledged to be moderated by cognitive reserve status in healthy aging (Franzmeier et al., 2017). Less is known regarding how different educational attainment might influence connectivity among middle-aged individuals. Yet, lower socioeconomic status, a proxy of educational level, has been related to less organized function brain networks in middle-aged adults (Chan et al., 2018). For this reason, it would be advisable for future studies to include subjects with lower levels of education in order to explore the scope of RNT-system segregation associations and possible interactions with educational level/cognitive reserve. Finally, fMRI and questionnaires of RNT were not undertaken at the same time, with an interval period around 12 and 18 months. Nonetheless, recent evidence suggests that RNT is a stable construct more linked to trait than to symptom severity (Hijne et al., 2020), particularly perseverative thinking, which is conceptualized as a content-independent measure of RNT (Ehring et al., 2011).

## Conclusion

System segregation, understood as the balance of between-network and within-network connectivity (Ewers et al., 2021) might be a biomarker of RNT among healthy middle-aged adults. Forthcoming studies should be designed to corroborate the present findings linking perseverative thinking and segregation of executive and salience networks; particularly among individuals at risk for cognitive decline.

## Data availability statement

The data analyzed in this study is subject to the following licenses/restrictions: data may be made available upon reasonable request, given appropriate ethical and data protection approvals. Requests to access the datasets should be directed to https://bbhi.cat/en/contact/.

## Ethics statement

The studies involving human participants were reviewed and approved by This study was carried out in accordance with the recommendations of the “Unió Catalana d’Hospitals” and received approval. The patients/participants provided their written informed consent to participate in this study.

## Author contributions

CS-P contributed to conceptualization, data curation, formal analysis and writing (original draft). GC, LM-P, JS, and JT contributed to resources and data curation. NM and ÁP-L contributed to conceptualization and writing (review and editing). MC-T contributed to software, formal analysis and visualization. NB contributed to resources and data curation. DB-F contributed to conceptualization, supervision, writing (review and editing) and project administration. All authors contributed to the article and approved the submitted version.

## Funding

The study was supported by a grant from the Spanish Ministry of Science, Innovation and Universities (MICIU/FEDER; grant number RTI2018-095181-B-C21) and an ICREA Academia 2019 grant award to DB-F. This work received partial funding from “La Caixa” Foundation (grant number LCF/PR/PR16/11110004), and from Institut Guttmann and Fundació Abertis. NM was supported by a Senior Fellowship from the Alzheimer’s Society (AS-SF-15b-002).

## Conflict of interest

ÁP-L is listed as an inventor on several issued and pending patents on the real-time integration of noninvasive brain stimulation with electroencephalography and magnetic resonance imaging. He is co-founder of Linus Health and TI Solutions AG; and serves on the scientific advisory boards for Starlab Neuroscience, Magstim Inc., Nexstim, and MedRhythms.

The remaining authors declare that the research was conducted in the absence of any commercial or financial relationships that could be construed as a potential conflict of interest.

## Publisher’s note

All claims expressed in this article are solely those of the authors and do not necessarily represent those of their affiliated organizations, or those of the publisher, the editors and the reviewers. Any product that may be evaluated in this article, or claim that may be made by its manufacturer, is not guaranteed or endorsed by the publisher.

## Footnotes

^1^FSL; version 5.0.11; https://fsl.fmrib.ox.ac.uk/fsl/fslwiki/

^2^version 6.0; https://surfer.nmr.mgh.harvard.edu

^3^SPM12; https://www.fil.ion.ucl.ac.uk/spm/
